# The realistic positioning of UVA1 phototherapy after 25 years of clinical experience and the availability of new biologics and small molecules: a retrospective clinical study

**DOI:** 10.3389/fmed.2023.1295145

**Published:** 2023-11-21

**Authors:** Piergiacomo Calzavara-Pinton, Luca Bettolini, Francesco Tonon, Mariateresa Rossi, Marina Venturini

**Affiliations:** ^1^Department of Dermatology, University of Brescia, Brescia, Italy; ^2^Department of Dermatology, ASST Spedali Civili di Brescia, Brescia, Italy

**Keywords:** ultraviolet A1, phototherapy, atopic dermatitis, morphea, GVHD

## Abstract

**Background:**

Since the early 1990s, Ultraviolet (UV) A1 phototherapy has been described as an effective and safe treatment of a multitude of skin disorders. However, after 30 years, its use has remained limited to few dermatological centers.

**Objective:**

To analyze the changes over the years and the current position of UVA1 phototherapy through a Real-World Evidence (RWE) study at a single tertiary referral center.

**Methods:**

We reviewed the medical files of 740 patients treated between 1998 and 2022. Treatment results were collected, efficacy was assessed by a grading scale and acute adverse effects were registered.

**Results:**

We treated patients with 26 different diseases. We registered marked improvement (MI) or complete remission (CR) in 42.8% of patients with morphea, 50% with Urticaria Pigmentosa, 40.7% with Granuloma annulare and 85.7% with skin sarcoidosis. Good results were obtained also in the treatment of chronic Graft Versus Host Disease (GVHD), Eosinophilic Fasciitis, Sclero-atrophic Lichen, skin manifestations of systemic lupus erythematosus and psoriasis of HIV+ patients. Systemic Sclerosis, Romberg’s Syndrome, Bushke’s Scleredema, Nephrogenic Fibrosing Dermopathy, REM Syndrome, Follicular Mucinosis, Pretibial Myxedema, Scleromyxedema, pemphigus foliaceus, chronic cutaneous lupus erythematosus, erythroderma of Netherton Syndrome and Necrobiosis Lipoidica were no or poorly responsive. In clinical indications where UVA1 was used as a second line phototherapy after narrow-band (NB)-UVB, we saw good MI or CR rates in Mycosis Fungoides (57% of patients), Atopic Dermatitis (33.9%), Pitiryasis Lichenoides chronica (50%), Pityriasis Lichenoides et varioliformis acute (75%) and Lymphomatod Papulosis (62.5%). Short-term adverse events were uncommon and mild.

**Conclusion:**

Over the past decade, the annual number of treated patients has progressively declined for several reasons. Firstly, UVA1 phototherapy has taken a backseat to the cheaper and more practical NB-UVB phototherapy, which has proven effective for common indications. Secondly, the emergence of new, safe, and effective drugs for conditions such as atopic dermatitis, GVHD, and connective tissue disorders. Finally, our research has shown that UVA1 therapy is often ineffective or minimally effective for some rare diseases, contrary to previous case reports and small case series. Nonetheless, UVA1 continues to be a valuable treatment option for patients with specific skin disorders.

## 1 Introduction

Ultraviolet (UV) A1 (340–400 nm) radiation has two main photobiological peculiarities which differentiate it from UV wavebands with shorter wavelengths. First, it causes oxidative photochemical damages in cell structures with a limited contribute of anaerobic reactions and, second, it penetrates into the dermis targeting not only epidermal cell populations but also dermal resident and trafficking immune-competent cell populations, mastocytes and fibroblasts.

UVA1 phototherapy was introduced into dermatological clinical practice in the early 1990s (1). However, after 30 years (2), its use is still limited to a few dermatological centers in Europe, Japan and the US (3) although many studies have demonstrated its efficacy in a multitude of skin conditions.

Unfortunately, however, the quality of these studies is limited. Randomized clinical trials (RCTs) have studied only a few clinical indications, i.e., Morphea, Systemic Lupus Erythematosus (SLE), Urticaria Pigmentosa, Atopic Dermatitis, Dyshidrotic Eczema and subacute prurigo and they also had a high risk of bias with several main limitations: low number of enrolled patients, different treatment protocols and short follow-up (1, 3, 4).

For the other clinical indications, the strength of evidence is even poorer because only isolated case reports, uncontrolled pilot studies of small case series and retrospective studies are available (1, 3–6).

Furthermore, there is an overlap of indications with NB-UVB and, in the absence of direct comparative studies, UVA1 should be considered a second-line phototherapy because the equipment is more expensive, has a higher electricity consumption and each individual exposure is more time consuming.

Moreover, comparative studies with drug treatments, including the most recent immunotherapies that, unlike phototherapies, allow for a long-term control of selected diseases, have never been done.

With the aim of clarifying the current role of UVA1 phototherapy and the changes of its clinical uses over the past 25 years, we report here a retrospective analysis of the medical records of the largest case series ever published, 740 patients, who underwent at least a treatment cycle with UVA1 phototherapy in the years 1998–2022.

## 2 Materials and methods

This is a single-center retrospective and observational study. We reviewed the medical files of 740 patients who underwent at least a treatment cycle with medium-dose (40–60 J/cm2) UVA1 phototherapy from 1998 to 2022 at the Photodermatology Unit of the ASST Spedali Civili and University of Brescia, a tertiary referral center in Northern Italy.

We enrolled patients for whom approved topical or systemic therapies were unlikely to be effective or were at high risk of adverse effects, were discontinued because of the development of toxicity, or were contraindicated because of co-morbidities or concurrent therapies. UVA1 was a first line phototherapy for patients with Morphea, disseminated Granuloma Annulare, Eosinophilic Fasciitis, Necrobiosis Lipodica, Buschke Scleredema, Romberg’s Disease, Systemic Sclerosis, Pre-tibial Myxedema, Scleromyxedema, SLE, Chronic Cutaneous Lupus Erythematosus (CCLE), Urticaria Pigmentosa (UP), Psoriasis and pemphigus foliaceus in HIV+ patients, REM Syndrome, Follicular Mucinosis, Nephrogenic Fibrosing Dermopathy and chronic Graft versus Host Disease (GVHD). It was used, as a second line phototherapy, after NB-UVB phototherapy, for patients with Atopic Dermatitis, Mycosis Fungoides, Pityriasis Lichenoides Chronica (PLC), Pityriasis Lichenoides et Varioliformis Acuta (PLEVA), Lymphomatoid Papulosis (LyP), Sclero-Atrophic Lichen and Erythroderma of Netherton Syndrome.

All subjects gave written informed consent prior to being treated.

The diagnosis was assessed visually in most patients and a biopsy for histological confirmation was taken only in selected cases.

Contraindications to the treatment were pregnancy or lactation, any active systemic infectious disease, other inflammatory, infectious or neoplastic skin diseases in the area treated, history of photosensitivity, use of immunosuppressive or photosensitizing drugs, and history or indicators of poor compliance.

The study protocol was reviewed and approved by the Ethics Commission of ASST-Spedali Civili di Brescia under approval number 4710. It was carried out in strict adherence to the principles outlined in the Declaration of Helsinki, ensuring the participants’ data confidentiality and their absolute right to withdraw from the study at any point.

### 2.1 Phototherapy units

**Figure 1:**
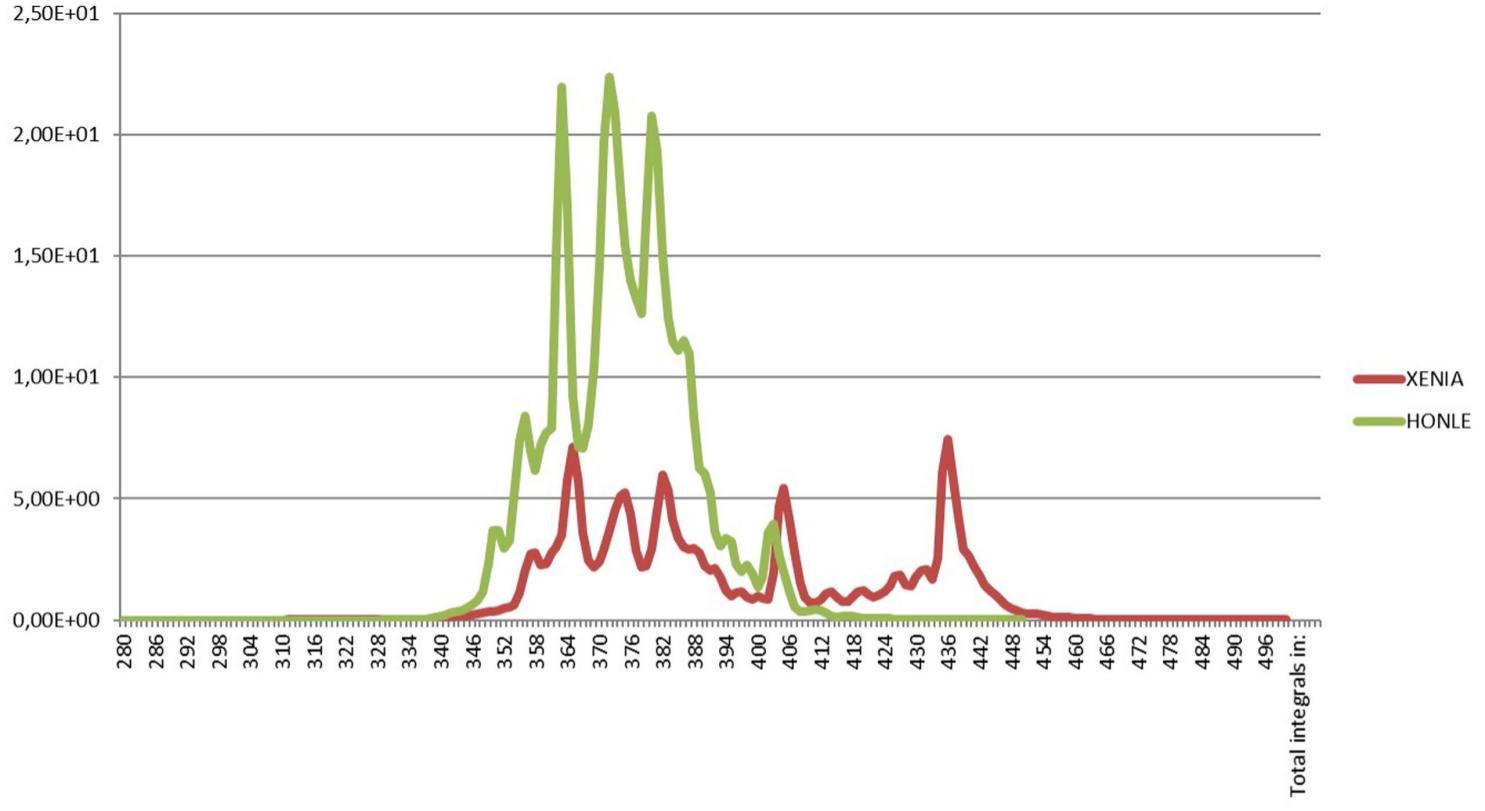
Emission spectrum of the Dermalight ultraA1 (Dr. Hoenle GmbH, Kaufering, Germany) whole body unit equipped with metal halide lamps that we used from 1998 to 2013 and emission spectrum of the MediSun Xenia (Schulze and Bohm Gmbh, Bruhl, Germany) with xenon lamps that we have been using since 2014. Both irradiation units have a UV emission strictly confined in the UVA1 range from 340 to 400 nm. The irradiances were 50.0 mW/cm2 and 14.3 mW/cm2 at skin level, respectively. Irradiance was measured with portable broadband UV meters (Waldmann) after calibration with a Macam SR 9910 spectroradiometer (Macam Photometrics Ltd., Livingston, United Kingdom).

We used from 1998 to 2013 a Dermalight ultraA1 (Dr. Hoenle GmbH, Kaufering, Germany) whole body units equipped with metal halide lamps and, since 2014 a MediSun Xenia (Schulze & Bohm Gmbh, Bruhl, Germany) with xenon lamps. As shown, in Figure 1, both irradiation units have a UV emission strictly confined in the UVA1 range from 340 to 400 nm but the irradiance was much higher with the Dermalight (50.0 mW/cm2 versus 14.3 mW/cm2 at skin level with the Xenia, respectively) and therefore the duration of exposure was approximately 3.5 times shorter (Figure 1). From an organizational point of view this means that fewer patients can be treated in the same amount of time and therefore that to treat the same number of patients the photodermatology center needs to be open longer. Irradiance was measured with portable broadband UV meters (Waldmann) after calibration with a Macam SR 9910 spectroradiometer (Macam Photometrics Ltd., Livingston, United Kingdom).

### 2.2 Treatment protocol

All patients treated with medium-dose UVA1 received a first dose of 30 J/cm2 and, if well tolerated, fixed daily exposures of 40–60 J/cm2 were delivered twice a week on non-consecutive days. In case of a phototoxic reaction, the daily dose was reduced by 10–20 J/cm2.

A low-dose protocol (10–20 J/cm2) was employed only for patients with SLE.

Treatments were continued until complete clearing was obtained, or until partial or no improvement was seen without further amelioration despite 6 additional treatments.

### 2.3 Study procedures

A standardized form was performed to collect the following data:

- Pre-treatment information: patients’ demographic data, baseline lesion characteristics and severity, histopathological findings (if present), and prior therapies and efficacy.
- Treatment parameters: light source, light dose, number of treatment cycles, mean irradiations per cycle, mean UVA1 dose per cycle, cumulative number of irradiations and cumulative light dose received by patients. Data are reported as mean ± standard deviation (m ± SD).
- Efficacy: it was assessed at the end of the treatment cycles on the basis of the clinical documentation by a grading scale with 7 grades: (−2) withdrawal within six irradiations for various non-treatment related reasons; (−1) aggravation; (0) no change; (1) slight improvement; (2) moderate improvement; (3) marked improvement; (4) complete remission (A).
- Adverse events: local phototoxic reactions were considered mild if temporary pinkish erythema was seen, moderate if the erythema was frankly red and self-resolving in few days and marked if persistent erythema with edema and/or erosions developed. Itching, pain and/or burning sensation were rated according to the following score: mild (therapy could be continued at the same dosage); moderate (irradiation was reduced); intense (treatment was stopped and then resumed or definitely).

## 3 Results

Fully evaluable clinical records of 740 patients were collected. They were suffering from 26 different skin conditions and the year of beginning of their first treatment is reported in Table 1.

**Table 1:** Number of patients undergoing a first treatment cycle per year.

Diagnosis	1998	1999	2000	2001	2002	2003	2004	2005	2006	2007	2008	2009	2010	2011	2012	2013	2014	2015	2016	2017	2018	2019	2020	2021	2022	total
Skin disorders with dermal lymphocytic inflammatory or tumoral infiltrates																										
Atopic dermatitis	7	10	10	9	11	6	14	12	5	5	11	7	10	8	10	11	8	9	8	3	1	2	2	1	3	183
Psoriasis					2	3				3					1						2					11
Pemphigus foliaceus				1																						1
Netherton Syndrome		1																								1
PLC					2		1	1	1		1								2			1	1			10
PLEVA					1		1		2	2		1				1		2				1		1		12
Lymphomatoid papulosis			1	1			2			1	1		1			1										8
Mycosis Fungoides	2	2	4	3	4	4	3	5	4	4	6	6	7	3	5	5	1	3	1		2		1	2	2	79
SCLE					1	2	2					1		3			1			1						11
CCLE					1	2	1	1																		5
Skin disorders with dermal mastocytic infiltrate																										
UP		3		1	1	3		2	1	2			1	2				1							1	18
Skin disorders with granulomatous dermal infiltrates																										
Granuloma annulare			2	3		1	1	1	3	2	2	2	1	5	5	2	3	3	1	4	3	2	4	2	2	54
Necrobiosis lipoidica					2	1	1		2																	6
Sarcoidosis														1	2			1			1		1	1		7
Skin disorders with dermal sclerosis, fibrosis or mucinosis																										
Morphea	6	5	10	11	6	7	3	7	9	7	8	11	7	5	8	11	9	6	8	10	10	10	9	10	8	201
Romberg’s syndrome							1	1	1			2														5
Systemic sclerosis						2	2	1																		5
Nephrogenic fibrosing dermopathy					1																					1
Scleroatrophic lichen						3	2	2	2	3	2	1	1	3	4	1	1	1	2	2			1			31
Eosinophilic fasciitis		1				1				1	1		1				1				1	1				8
Buschke’s scleredema								2		2			1													5
Pretibial myxoedema									1	2			1	1			1									6
Sclero-myxedema								2			1	2														5
REM									1																	1
Follicular mucinosis									1	1																2
GVHD		3	4	3	3	2	4	2	6	3	4	3	5	3	2	4	2	1	1	2		2	1	2	2	64
Total	15	25	31	32	35	37	38	39	39	38	37	36	36	34	37	36	27	27	23	22	20	19	20	19	18	740

CCLE, chronic cutaneous lupus erythematosus; NFD, nephrogenic fibrosing dermopathy; PLEVA, pityriasis lichenoides et varioliformis acuta; PLC, pityriasis lichenoides chronica; REM, reticular erythematous mucinosis; SLE, systemic lupus erythematosus; UP, urticaria pigmentosa.

**Figure 2:**
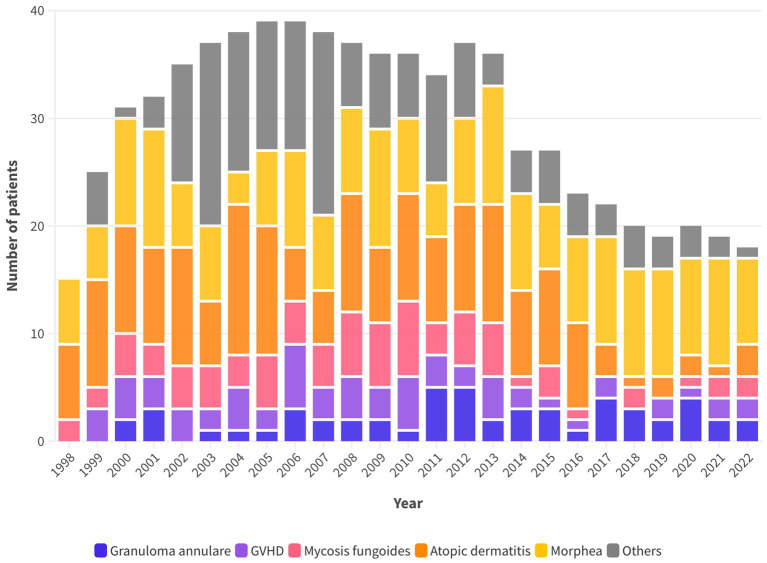
Annual number of patients who underwent a first therapeutic cycle with UVA1.

After the first years (1998–2001), the annual number of patients beginning the first treatment cycle ranged was quite stable ranging from 36 to 39 in the years 2002 to 2013. It began to decline in the year 2014, after replacing the irradiation unit with the metal halide lamps with the more practical and less expensive but also less powerful unit with Xenon lamps, the number of patients decreased. At the same time, some potential indications that were explored with disappointing results were abandoned (Figure 1). Figure 2 shows that the decline of the number of treated patients was particularly clear for patients with AD and Mycosis Fungoides after 2017, when biologics and other new drugs for their skin conditions became available in Italy while the number of patients with other indications remained rather stable over time.

Table 2 summarizes the clinical details and treatment results of patients affected by clinical indications with at least 5 treated patients: gender, age, number of treatment cycles, number of irradiations per treatment cycle, UVA1 dose of the exposure (J/cm2), cumulative UVA1 dose per treatment cycle (J/cm2) and cumulative number of irradiations and cumulative UVA1 dose (J/cm2) of all treatment cycles. The largest groups of treated patients were affected by Morphea (*n* = 201), Atopic Dermatitis (*n* = 183), Mycosis Fungoides (*n* = 79), GVHD (*n* = 64), Granuloma Annulare (*n* = 54), and Scleroatrophic Lichen (*n* = 31). The number of patients with Atopic Dermatitis and Mycosis Fungoides decreased after 2017 and 2014, respectively (Table 1 and Figure 2).

**Table 2:** Summary of the patients’ main clinical features and phototherapy results.

Diagnosis	No of patients	Gender [M (%)/F (%)]	Age (years) (range)	Treatment cycles	Irradiations per cycle	UVA1 dose of single exposure (J/cm^2^)	Cumulative UVA1 dose per cycle (J/cm^2^)	Cumulative number of irradiations	Cumulative UVA1 dose of all cycles (J/cm^2^)
Skin disorders with dermal lymphocytic inflammatory or tumoral infiltrates									
Atopic dermatitis	183	80/103	25.6 (6–52)	1.4 ± 0.7	29.6 ± 21.4	49.7 ± 9.4	1459.4 ± 999.5	41.0 ± 38.7	2103.8 ± 2024.0
Psoriasis of HIV+ patients	11	7/4	32.0 (22–48)	1.3 ± 0.9	27.1 ± 12.7	48.2 ± 12.0	1333.3 ± 643.7	37.6 ± 35.7	1851.7 ± 1788.5
PLC	10	7/3	28.4 (14–36)	1.0 ± 0.0	26.3 ± 14.8	61.1 ± 20.7	1595.7 ± 694.1	26.3 ± 14.8	1595.7 ± 694.1
PLEVA	12	8/4	30.1 (18–41)	1.1 ± 0.4	27.6 ± 16.9	54.7 ± 11.9	1649.3 ± 1349.0	29.3 ± 15.6	1735.0 ± 1288.9
Lymphomatoid papulosis	8	3/5	29.4 (16–52)	1.5 ± 0.5	25.1 ± 14.8	46.5 ± 5.3	1132.9 ± 632.6	28.7 ± 17.5	1532.9 ± 1140.8
Mycosis fungoides	79	51/28	46.3 (26–83)	1.5 ± 0.9	32.8 ± 13.7	62.6 ± 19.0	2048.7 ± 1049.1	49.0 ± 35.1	2048.7 ± 1049.1
SLE	11	1/10	31.5 (16–68)	1.8 ± 0.8	17.1 ± 11.6	17.7 ± 12.1	178.8 ± 22.8	60.8 ± 88.8	540.2 ± 463.4
CCLE	5	1/4	48.4 (31–74)	1.5 ± 0.8	19.0 ± 4.6	41.8 ± 8.4	778.4 ± 169.1	41.8 ± 49.9	1831.2 ± 2331.1
Skin disorders with infiltrate of mastocytes									
UP	18	8/10	17.0 (8–27)	1.2 ± 0.4	23.5 ± 8.5	66.5 ± 16.8	1266.7 ± 771.4	29.0 ± 14.6	1816.2 ± 893.3
Skin disorders with granulomatous dermal infiltrates									
Granuloma annulare	54	25/29	43.8 (23–76)	1.7 ± 1.7	26.8 ± 12.8	52.8 ± 13.7	1590.8 ± 937.5	47.5 ± 45.8	2514.6 ± 2457.2
Necrobiosis lipoidica	6	1/5	48.7 (31–71)	1.6 ± 1.5	24.9 ± 7.9	51.2 ± 5.2	1295.1 ± 406.3	40.3 ± 41.3	2270.0 ± 2784.3
Sarcoidosis	7	5/2	41.4 (29–61)	1.7 ± 1.2	36.7 ± 14.6	67.6 ± 20.6	2611.0 ± 1600.0	60.0 ± 41.6	4353.3 ± 3417.3
Skin disorders with dermal sclerosis. Fibrosis or mucinosis									
Morphea	201	123/79	37.8 (26–82)	1.8 ± 1.5	32.2 ± 20.0	54.6 ± 13.5	1688.7 ± 1188.9	56.0 ± 49.8	3115.7 ± 2955.9
Romberg’s syndrome	5	1/4	28.9 (19–53)	1.0 ± 0.0	18.6 ± 6.2	61.4 ± 15.1	1028.1 ± 169.5	18.6 ± 6.2	1028.1 ± 169.5
Systemic sclerosis	5	0/5	44.3 (31–73)	1.4 ± 1.0	46.1 ± 25.0	47.6 ± 13.3	1561.1 ± 1516.0	60.6 ± 37.8	2994.0 ± 2094.0
Sclero-atrophic lichen	31	15/16	39.6 (33–81)	1.7 ± 1.5	30.1 ± 13.2	60.4 ± 19.4	1664.8 ± 793.8	50.4 ± 52.0	3101.3 ± 2675.6
Eosinophilic fasciitis	8	1/7	51.4 (40–76)	1.3 ± 0.5	67.6 ± 44.9	58.8 ± 20.3	5141.3 ± 4632.1	82.0 ± 49.5	5857.5 ± 4377.3
Buschke’s scleredema	5	4/1	62.4 (56–80)	2.8 ± 1.6	45.9 ± 34.6	50.6 ± 6.4	2970.3 ± 1508.2	90.8 ± 35.5	6005.5 ± 2360.4
Pretibial myxedema	6	5/1	53.1 (39–65)	3.5 ± 2.1	34.5 ± 16.3	47.6 ± 0.6	1,665 ± 756.6	105.7 ± 17.0	5025.0 ± 883.9
Scleromyxedema	5	2/3	43.4 (27–73)	2.3 ± 1.0	27.3 ± 12.6	51.6 ± 8.5	1343.7 ± 479.9	79.5 ± 67.3	3323.6 ± 2274.4
GVHD	64	28/36	29.9 (6–49)	1.8 ± 1.4	39.6 ± 35.4	54.1 ± 16.4	2102.3 ± 1845.9	71.1 ± 75.7	3688.1 ± 3970.3

Data of treatments are reported as mean ± standard deviation (m ± SD) (only diseases with at least 5 treated patients are listed).

CCLE, chronic cutaneous lupus erythematosus; PLEVA, pityriasis lichenoides et varioliformis acuta; PLC, pityriasis lichenoides chronica; REM, reticular erythematous mucinosis; SLE, systemic lupus erythematosus; UP, urticaria pigmentosa.

The mean number of treatment cycles ranged from 1.0 ± 0.0 for PLC and Romberg’s Syndrome to 2.8 ± 1.6 for Buschke Scleredema.

The mean number of exposures per treatment cycle ranged from 18.6 ± 6.2 for Romberg’s Syndrome to 67.6 ± 44.9 for eosinophilic fasciitis.

The mean UVA1 dose per exposure was 53.3 ± 11.0 J/cm2 with the lowest dose (17.7 ± 12.1 J/cm2) for SLE and the highest (58.8 ± 20.3 J/cm2) for eosinophilic fasciitis.

The mean total UVA1 dose per treatment cycle ranged from 178.8 ± 22.8 J/cm2 for SLE to 5141.3 ± 4632.1 J/cm2 for eosinophilic fasciitis.

The mean cumulative number of irradiations of all their treatment cycles was the lowest (26.3 ± 14.8) for PLC and the highest (105.7 ± 17.0) for pretibial myxedema and the mean cumulative UVA1 dose, was the lowest 540.2 ± 463.4 J/cm2 for SLE and the highest 6005.5 ± 2360.4 J/cm2 for Buschke Scleredema (Table 2).

In Table 3 we have described the clinical results in the treatment of skin disorders with at least 5 treated patients.

**Table 3:** Clinical outcome of UVA1 phototherapy.

Diagnosis	Number of patients	Grading score of the treatment outcome. *n* (%)						
		-2	-1	0	1	2	3	4
	*n*	*n* (%)	*n* (%)	*n* (%)	*n* (%)	*n* (%)	*n* (%)	*n* (%)
Skin disorders with dermal lymphocytic inflammatory or tumoral infiltrates								
Atopic dermatitis	183	16 (8.7)	10 (5.5)	22 (12.0)	34 (18.6)	39 (21.3)	36 (19.7)	26 (14.2)
Psoriasis of HIV + patients	11	0	1 (9.1)	2 (18.2)	2 (18.2)	0	3 (27.3)	3 (27.3)
PLC	10	1 (10)	0	0	1 (10)	3 (30)	2 (20)	3 (30)
PLEVA	12	0	0	0	1 (8.3)	2 (16.7)	4 (33.3)	5 (41.7)
Lymphomatoid papulosis	8	0	0	1 (12.5)	1 (12.5)	1 (12.5)	2 (25)	3 (37.5)
Mycosis fungoides	79	0	2 (2.5)	8 (10.1)	12 (15.2)	12 (15.2)	21 (26.6)	24 (30.4)
SLE	11	2 (18.2)	2 (18.2)	1 (9.1)	2 (18.2)	4 (36.4)	0	0
CCLE	5	1 (20)	2 (40)	0	2 (40)	0	0	0
Skin disorders with infiltrate of mastocytes								
UP	18	2 (11.1)	2 (11.1)	1 (5.6)	2 (11.1)	2 (11.1)	4 (22.2)	5 (27.8)
Skin disorders with granulomatous dermal infiltrates								
Granuloma annulare	54	4 (7.4)	0	5 (9.3)	11 (20.4)	12 (22.2)	14 (25.9)	8 (14.8)
Necrobiosis lipoidica	6	0	0	4 (66.7)	2 (33.3)	0	0	0
Sarcoidosis	7	0	0	2 (28.6)	0	1 (14.3)	4 (57.1)	2 (28.6)
Skin disorders with sclerosis, fibrosis or mucinosis								
Morphea	201	6 (3.0)	6 (3.0)	22 (10.9)	37 (18.4)	44 (21.9)	38 (18.9)	48 (23.9)
Romberg’s syndrome	5	0	2 (40)	3 (60)	0	0	0	0
Systemic sclerosis	5	0	1 (20)	2 (40)	2 (40)	0	0	0
Sclero-atrophic lichen	31	0	0	0	1 (3.2)	3 (9.7)	15 (48.4)	12 (38.7)
Eosinophilic fasciitis	8	0	0	2 (25)	0	1 (12.5)	3 (37.5)	2 (25)
Buschke’s scleredema	5	0	0	2 (40)	1 (20)	2 (40)	0	0
Pretibial myxedema	6	0	0	3 (50)	3 (50)	0	0	0
Sclero-myxedema	5	0	1 (20)	3 (60)	1 (20)	0	0	0
GVHD	64	6 (9.4)	5 (7.8)	10 (15.6)	6 (9.4)	14 (21.9)	15 (23.4)	8 (12.5)

The descriptive documentation was carried out in written records. The efficacy of the phototherapy was assessed by a grading scale with 7 grades: (−2) withdrawal within six irradiations for various non-treatment related reasons; (−1) aggravation; (0) no change; (1) slight improvement; (2) moderate improvement; (3) marked improvement; (4) complete remission (A). CCLE, chronic cutaneous lupus erythematosus; PLEVA, pityriasis lichenoides et varioliformis acuta; PLC, pityriasis lichenoides chronica; REM, reticular erythematous mucinosis; SLE, systemic lupus erythematosus; UP, urticaria pigmentosa.

The rates of patients achieving a complete remission or marked improvement was 33.9% in Atopic Dermatitis, 54.6% in Psoriasis of HIV+ patients, 50.0% in PLC, 75% in PLEVA, 62.5% in Lymphomatoid Papulosis, 57.0% in Mycosis Fungoides, 40.7% in Granuloma Annulare, 86.3% in Sarcoidosis, 42.8% in Morphea, 87.1% in Scleroatrophic Lichen and 62.5% in Eosinophilic Fasciitis.

Patients with chronic GVHD (28 sclerodermoid, 14 lichenoid and 22 mixed) had skin lesions without a severe involvement of internal organs. The rate of patients with complete remission or marked improvement was 35.9 and 21.9% had a moderate improvement. The main clinical advantage for these patients was that they could reduce the dosage of immunosuppressive drugs thus improving or reducing the severity of drug adverse effects and opportunistic infections.

CR and MI were observed in 50.0% out of 18 patients with UP. However, the score was based on the reduction of itching while we never observed a significant improvement of pigmentary changes.

All 11 patients with SLE had photosensitivity. Four patients had a moderate improvement of the skin lesions, but amelioration of the systemic manifestations was never registered. Although we used a low-dose protocol (10–30 J/cm2), 4 patients discontinued the treatment because of discomfort or worsening of the skin manifestations (Table 3). Results in CCLE were disappointing with only 2 patients achieving a mild improvement.

Patients with NLD, Romberg’s Syndrome, Systemic Sclerosis, Buschke’s Scleredema, and Scleromyxedema never achieved a complete or marked remission (Table 3). In addition, we treated a HIV+ patient suffering from a pemphigus foliaceus and a patient with Netherton disease. In both patients, the skin manifestations improved but there was a quick relapse after discontinuation. Patients with REM Syndrome (1 patient), Follicular Mucinosis (2 patients) and Nephrogenic Fibrosing Dermopathy (1 patient) did not improve.

A total of 34 (4.6%) patients discontinued the treatment due to worsening disease. The relative rate for each disorder was 5.5% of patients with atopic dermatitis, 9.1% in HIV+ psoriatic patients, 40% with CCLE, 18.2% with SCLE, 2.5% with mycosis fungoides, 11.1% with UP, 3% with morphea, 20% with systemic sclerosis, 40% with Romberg’s syndrome, 20% with scleromyxedema, and 7.8% with GVHD.

Thirty-eight (5.1%) patients discontinued the treatment cycle within 6 exposures for reasons unrelated to the treatment like the difficulties in getting organized to reach our center regularly, the lack of a caregiver, problems or work etc.

UVA1 phototherapy was always well-tolerated with only minor acute side effects, e.g., episodes of mild sunburns, itching and dryness of the skin that were easily controlled with the reduction of the UVA1 dose and the application of emollient creams. No patient discontinued the treatment because of adverse effects. Long-term exposure to UVA radiation can theoretically increase the risk of skin cancer but the incidence, if any, was never assessed and the present study was not designed to investigate it. However, we previously reported two cases of Merkel cell carcinoma arising in patients with drug-induced immunosuppression (7).

## 4 Discussion

The present RWE retrospective study demonstrated that UVA1 phototherapy is an effective and safe treatment option for various skin conditions. However, we have noted a decreasing trend in the number of patients who started a first cycle of therapy (Figure 2 and Table 1): after the first years (1998–2001) in which we familiarized with the new technique, the number of patients per year was quite stable ranging from 36 to 39 in the years 2002 to 2013 but, afterwards it started to decline to 18–20 since 2018 to 2022.

Looking at Table 1 and Figure 2, we see that the number of patients with a few indications remained approximately the same, e.g., morphea, sarcoidosis, granuloma annulare, chronic GVHD, UP, eosinophilic fasciitis and scleroatrophic lichen, over the years while it decreased for others. Indeed, after the publication of studies concluding that UVA1 phototherapy is not superior to the cheaper and more practical NB-UVB phototherapy, the treatment of PLC, PLEVA, and Mycosis Fungoides was restricted to only patients poorly responsive or with contraindications to NB-UVB phototherapy. In addition, in the case of atopic dermatitis, the number of patients decreased further after 2017, the year in which new effective therapeutic options such as anti-IL4 and IL13 biologics and JAK inhibitors for atopic became available. These drugs have set a new paradigm of AD treatment that is not only a complete or near-complete clearance at the end of treatment, but also a durable remission over time with the prevention of acute flares. In this new treatment landscape, we have limited UVA1 phototherapy to NB-UVB resistant patients who had a moderate disease and a treatment course characterized by long periods of remission after treatment (8).

Furthermore, the use for some uncommon clinical indications, i.e., Necrobiosis Lipoidica, Romberg’s Disease, Systemic Sclerosis, Pretibial Myxedema, Scleromyxedema, CCLE and others, was discontinued if, unlike previous reports we had observed disappointing results (see the detail later).

We therefore compared our data from RWE experience with the evidences from the data in the treatment of common dermatoses from studies in the literature. Atopic Dermatitis was the first target of UVA1 phototherapy (2) because there are several biological effects that can contribute to its improvement: suppression of the antigen-presenting function of Langerhans cells, induction of apoptosis of infiltrating T-cells, thickening of the stratum corneum, decreased susceptibility to pathogens, namely *Staphylococcus aureus* and Pityrosporum orbiculare (9). All randomized clinical studies have reported a significant improvement in symptoms such as pruritus and skin inflammation (2, 10–12) and the medium dose (40–60 J/cm2) regimen seems preferable because it is more effective than the low-dose regimen (10) and equally effective than the high dose regimen (12).

Other RCTs have demonstrated that medium dose UVA1 is not more effective than NB-UVB phototherapy (13–15). Therefore, in daily clinical practice, it is reasonable that NB-UVB is considered the first-line phototherapy with medium-dose phototherapy as second line treatment for patients that do not tolerate or are not responsive to NB-UVB.

This criterion of enrollment can explain why our results with UVA1 phototherapy are good but apparently lower to previous RCTs in which “naïve” patients were enrolled (2, 10–15).

Also in the case of PLC, PLEVA, LyP, and Mycosis Fungoides we have progressively used UVA1 phototherapy to only patients who were not candidates for, or not responsive to, standard NB-UVB phototherapy (16–19), PUVA therapy or drug therapies.

Despite these restrictive eligibility criteria, the therapeutic results in our patients with PLC, PLEVA and LyP was still very good with 50, 75 and 62.5% CR or MI, respectively, thus confirming the results of previous reports (20, 21). Furthermore, in most cases, only one therapeutic cycle gave a persistent remission (Table 2).

In patients with Mycosis Fungoides stages Ia, Ib, and IIa, the therapeutic response was relatively very good with 57% MI or CR although the results were worse than reported in previous clinical studies (22–24) in which, however, UVA1 phototherapy was used in patients regardless of the response to NB-UVB phototherapy and PUVA therapy.

NB-UVB is certainly the first line phototherapy for psoriasis. It has been demonstrated that UVA1 phototherapy has a lower efficacy with only a partial improvement of the histological parameters (25). However, we have used UVA1 in psoriatic patients with HIV infection (26) because immunotherapies are contraindicated and UVB irradiation activates HIV in human skin (27). Therapeutic results were good with 6 out of 11 patients achieving a MI or CR.

We also treated an HIV+ patient who was suffering from pemphigus foliaceus. However, the Improvement was mild and transitory with a quick relapse at discontinuation.

The improvement was nearly complete, but the relapse was quick at discontinuation also in a patient with erythroderma due to Netherton Syndrome (28).

A few RCTs have found that low-dose UVA1 phototherapy significantly reduced constitutional symptoms, joint pain, rashes, and the systemic lupus activity measures of SLE patients, and these beneficial effects were persistent at a follow-up of 3.4 years (29, 30).

We have used low-dose UVA1 phototherapy in SLE patients with cutaneous lesions and moderate systemic involvement with the aim to improve skin and systemic symptoms and reduce systemic toxicity by immunosuppressive drugs. A moderate improvement of skin lesions was seen in 4/11 (36.4%) patients but we never registered an amelioration of the systemic manifestations and we never could reduce the systemic corticosteroid dosage. In addition, two (18.2%) patients had a worsening of their cutaneous and systemic disease. The rationale of use of low doses of UVA − 1 is based on findings.

that it modulates Th1/Th2 and Tc1/Tc2 balances, reduces B cell activity, prevents the suppression of cell-mediated immunity and impairs an epigenetic progression toward SLE. In addition, UVA-1 seems to be effective in reducing anti-phospholipid acids and this could be of help in pregnant patients with lupus and anti-cardiolipin antibodies (29, 30).

Based on the encouraging results of a previous study (31), we treated 5 patients with (discoid lupus) with CCLE with drug intolerance or eye and/or liver toxic damage by oral chloroquine. However, again, results were disappointing.

UVA1 phototherapy has gained space as a first-line treatment of urticaria pigmentosa (32, 33) because evidences of the efficacy of NB-UVB phototherapy are limited and with poor quality (34). Moreover, it seems preferable to PUVA therapy in these patients that are young and therefore at high risk of long-term toxicity by psoralens. Our results were positive on itching and urticarial flares with 9 out of 18 (50%) patients attaining CR or MI whereas skin pigmentary lesions did not improve.

Other disorders for which UVA1 phototherapy may be considered first-line phototherapy are characterized by dermal granulomatous infiltrates or dermal sclerosis.

We have obtained overall very positive results in the treatment of 54 patients with widespread Granuloma Annulare and we obtained a MI or CR in 25.9 and 14.8% of patients, respectively. In addition, unlike a previous paper (35), the therapeutic result was quite persistent and only a small number of our patients needed more treatment cycles (mean ± SD: 1.7 ± 1.7).

In a case report (36), a patient with a chronic ulcerating Necrobiosis Lipoidica had a dramatic response and in a small case series of six patients, three had a moderate improvement or a resolution (37). However, we have treated 6 patients without improvement in 4 and only a minimal improvement in 2.

We can hypothesize that the difference of results from what was observed in the treatment of Granuloma Annulare, could be explained by the presence of cicatricial and atrophic areas in NLD that may hinder improvements and make clinical evaluation of the result more difficult. The treatment of seven patients with cutaneous sarcoidosis was instead very successful and the positive result is consistent with the results of 2 previous case reports (38, 39).

Given the ability of UVA1 phototherapy to inhibit *T*-cells and the collagen degradation and synthesis (40) several studies evaluated its use in the treatment of morphea and reported an improvement in skin thickness, as well as an improvement in symptoms such as pruritus and skin tightness (41) and the clinical improvement was confirmed by ultrasonographic morphological analysis (42). Our results in a very large group of 201 patients were also very good with only a minority of patients who did not improve (22; 10.9%) or had a progression (6; 3.0%) of the disease.

In our daily clinical practice, we have delivered medium-dose UVA1 because two RCTs have demonstrated that it is more effective than low-dose UVA1 and NB-UVB (43, 44). Similarly to the results of a previous preliminary study (45), medium-dose UVA1 phototherapy in our hands was an effective and well-tolerated treatment option for 31 patients with extragenital sclero-atrophic lichen with a complete or marked softening and re-pigmentation of the affected skin in 12 (38.7%) and 16 (51.6%) patients, respectively. The treatment was also successful in 2 patients with bullous lichen sclerosus thus confirming a previous case report (46).

Results with Eosinophilic fasciitis were encouraging with CR or MI in 5 out of 8 treated patients. These results are in general agreement with the findings of a previous case series of 8 patients (47).

The treatment of patients with lichenoid and sclerodermoid GVHD was limited to cases with prevalent skin involvement and mild disease of internal organs with the main goal to avoid or reduce the chronic use of systemic glucocorticosteroids and other immunosuppressants (48–50). The reasons for treating patients with UVA1 instead of photopheresis, a well-known and effective light-based treatment for cGVHD (51), were different: some patients had received no or moderate improvement with one or more previous treatment cycles with photopheresis, others lived far from a with photopheresis center and in some others it was particularly difficult to access a venous access.

In addition, the use of UVA1 was carefully evaluated in each individual patient because it has a carcinogenic potential, we treated a series of 64 cases and we had a MI and CR in 15 (23.4%) and 8 (12.5%) patients, respectively.

Unlike previous papers of small case series or case-reports, the treatment of systemic sclerosis (52, 53), facial hemiatrophy (Parry-Romberg Syndrome) (54), nephrogenic fibrosing dermopathy (55) and Buschke’s Scleredema (56–58), Pretibial myxedema (59) and other mucinoses, e.g., idiopathic follicular mucinosis (60, 61) and reticular erythematous mucinosis (REM) syndrome (62, 63) was unsuccessful.

The reasons for these discrepancies are the lack of large studies, individual variability in response, and the fact that these publications almost always reported successful outcomes, but this may be due to authors choosing not to submit negative observations, or, if they do, risk not being accepted for publication. However, the small number of enrolled patients in the present and previous studies is a very relevant limitation to the knowledge of the therapeutic potential of UVA1 phototherapy of these rare disorders.

In conclusion, the present RWE experience of 25 years on a very large number of cases allows to better understand the possible clinical indications although the retrospective design of the study remains a main limitation.

UVA1 phototherapy should be used as first line therapy only when efficacy and/or safety are superior to NB-UVB phototherapy and drug treatment options.

The number of clinical indications and patients treated has therefore decreased over time but UVA1 remains a first-line treatment for some skin disorders such as UP, morphea, eosinophilic granuloma, disseminated granuloma annulare, sclero-atrophic lichen and chronic GVHD. It can also be useful in atopic dermatitis, PLC, PLEVA, LyP, skin Sarcoidosis, and Mycosis Fungoides that are resistant to NB-UVB phototherapy and drug treatment options. Unfortunately, we could not confirm the efficacy for a few uncommon clinical indications that are supported only by low-quality studies on small series or case reports.

## Data availability statement

The raw data supporting the conclusions of this article will be made available by the authors, without undue reservation.

## Ethics statement

The studies involving humans were approved by the Ethics commission of ASST- Spedali Civili di Brescia, approval number 4710. The studies were conducted in accordance with the local legislation and institutional requirements. The participants provided their written informed consent to participate in this study.

## Author contributions

PC-P: Conceptualization, Methodology, Project administration, Writing – original draft. LB: Data curation, Methodology, Writing – original draft. FT: Writing – review & editing. MR: Formal analysis, Writing – review & editing. MV: Conceptualization, Writing – review & editing.

## References

[1] Krutmann J Schöpf E. High-dose-UVA1 phototherapy: a novel and highly effective approach for the treatment of acute exacerbation of atopic dermatitis. Acta Derm Venereol Suppl. (1992) 176:120–2. PMID: 1476022

[2] Krutmann J Czech W Diepgen T Niedner R Kapp A Schöpf E. High-dose UVA1 therapy in the treatment of patients with atopic dermatitis. J Am Acad Dermatol. (1992) 26:225–30. doi: 10.1016/0190-9622(92)70031-a, PMID: 1552057

[3] Kerr AC Ferguson J Attili SK Beattie PE Coleman AJ Dawe RS. Ultraviolet A1 phototherapy: a British Photodermatology group workshop report. Clin Exp Dermatol. (2012) 37:219–26. doi: 10.1111/j.1365-2230.2011.04256.x 22277060

[4] Connolly KL Griffith JL McEvoy M Lim HW. Ultraviolet A1 phototherapy beyond morphea: experience in 83 patients. Photodermatol Photoimmunol Photomed. (2015) 31:289–95. doi: 10.1111/phpp.12185 26052743

[5] Rombold S Lobisch K Katzer K Grazziotin TC Ring J Eberlein B. Efficacy of UVA1 phototherapy in 230 patients with various skin diseases. Photodermatol Photoimmunol Photomed. (2008) 24:19–23. doi: 10.1111/j.1600-0781.2008.00328.x 18201353

[6] Ronen S Ramot Y Zlotogorski A Shreberk-Hassidim R. Efficacy of ultraviolet A1 phototherapy for inflammatory, sclerotic and neoplastic dermatological diseases: a 10-year tertiary referral center experience. Photodermatol Photoimmunol Photomed. (2023) 39:256–62. doi: 10.1111/phpp.12833 36052749

[7] Calzavara-Pinton P Monari P Manganoni AM Ungari M Rossi MT Gualdi G. Merkel cell carcinoma arising in immunosuppressed patients treated with high-dose ultraviolet A1 (320-400 nm) phototherapy: a report of two cases. Photodermatol Photoimmunol Photomed. (2010) 26:263–5. doi: 10.1111/j.1600-0781.2010.00529.x 21175855

[8] Rossi M Damiani C Arisi M Tomasi C Tonon F Venturini M. Definition of the clinical characteristics of patients with moderate and severe atopic dermatitis for whom narrow-band UVB (NB-UVB) and medium-dose UVA1 phototherapies are still valuable treatment options at the age of biologics. J Clin Med. (2023) 12:3303. doi: 10.3390/jcm12093303 37176743 PMC10179382

[9] Grundmann SA Beissert S. Modern aspects of phototherapy for atopic dermatitis. J Allergy. (2012) 2012:121797. doi: 10.1155/2012/121797, PMID: 22220185 PMC3246755

[10] Kowalzick L Kleinheinz A Weichenthal M Neuber K Köhler I Grosch J. Low dose versus medium dose UV-A1 treatment in severe atopic eczema. Acta Derm Venereol. (1995) 75:43–5. doi: 10.2340/00015555754345, PMID: 7538257

[11] Krutmann J Diepgen TL Luger TA Grabbe S Meffert H Sönnichsen N. High-dose UVA1 therapy for atopic dermatitis: results of a multicenter trial. J Am Acad Dermatol. (1998) 38:589–93. doi: 10.1016/s0190-9622(98)70123-9 9555799

[12] Tzaneva S Seeber A Schwaiger M Hönigsmann H Tanew A. High-dose versus medium-dose UVA1 phototherapy for patients with severe generalized atopic dermatitis. J Am Acad Dermatol. (2001) 45:503–7. doi: 10.1067/mjd.2001.114743 11568738

[13] Legat FJ Hofer A Brabek E Quehenberger F Kerl H Wolf P. Narrowband UV-B vs medium-dose UV-A1 phototherapy in chronic atopic dermatitis. Arch Dermatol. (2003) 139:223–4. doi: 10.1001/archderm.139.2.223 12588233

[14] Majoie IM Oldhoff JM van Weelden H Laaper-Ertmann M Bousema MT Sigurdsson V. Narrowband ultraviolet B and medium-dose ultraviolet A1 are equally effective in the treatment of moderate to severe atopic dermatitis. J Am Acad Dermatol. (2009) 60:77–84. doi: 10.1016/j.jaad.2008.08.048 19103360

[15] Gambichler T Othlinghaus N Tomi NS Holland-Letz T Boms S Skrygan M. Medium-dose ultraviolet (UV) A1 vs. narrowband UVB phototherapy in atopic eczema: a randomized crossover study. Br J Dermatol. (2009) 160:652–8. doi: 10.1111/j.1365-2133.2008.08984.x 19120333

[16] Pavlotsky F Baum S Barzilai A Shpiro D Trau H. UVB therapy of pityriasis lichenoides--our experience with 29 patients. J Eur Acad Dermatol Venereol. (2006) 20:542–7. doi: 10.1111/j.1468-3083.2006.01531.x 16684281

[17] Ersoy-Evans S Hapa AA Boztepe G Sahin S Kölemen F. Narrowband ultraviolet-B phototherapy in pityriasis lichenoides chronica. J Dermatolog Treat. (2009) 20:109–13. doi: 10.1080/09546630802449088, PMID: 19016063

[18] Maranda EL Smith M Nguyen AH Patel VN Schachner LA Joaquin JJ. Phototherapy for Pityriasis Lichenoides in the pediatric population: a review of the published literature. Am J Clin Dermatol. (2016) 17:583–91. doi: 10.1007/s40257-016-0216-2, PMID: 27502793

[19] Trautinger F. Phototherapy of cutaneous T-cell lymphomas. Photochem Photobiol Sci. (2018) 17:1904–12. doi: 10.1039/c8pp00170g 30325389

[20] Pinton PC Capezzera R Zane C De Panfilis G. Medium-dose ultraviolet A1 therapy for pityriasis lichenoides et varioliformis acuta and pityriasis lichenoides chronica. J Am Acad Dermatol. (2002) 47:410–4. doi: 10.1067/mjd.2002.122199 12196751

[21] Calzavara-Pinton P Venturini M Sala R. Medium-dose UVA1 therapy of lymphomatoid papulosis. J Am Acad Dermatol. (2005) 52:530–2. doi: 10.1016/j.jaad.2004.09.036, PMID: 15761440

[22] Olek-Hrab K Silny W Dańczak-Pazdrowska A Osmola-Mańkowska A Sadowska PA Polańska A. Ultraviolet A1 phototherapy for mycosis fungoides. Clin Exp Dermatol. (2013) 38:126–30. doi: 10.1111/ced.12001 23082901

[23] Adışen E Tektaş V Erduran F Erdem Ö Gürer MA. Ultraviolet A1 phototherapy in the treatment of early mycosis Fungoides. Dermatology. (2017) 233:192–8. doi: 10.1159/000458149 28441652

[24] Zane C Leali C Airò P De Panfilis G Pinton PC. “high-dose” UVA1 therapy of widespread plaque-type, nodular, and erythrodermic mycosis fungoides. J Am Acad Dermatol. (2001) 44:629–33. doi: 10.1067/mjd.2001.110896 11260537

[25] Silpa-Archa N Pattanaprichakul P Charoenpipatsin N Jansuwan N Udompunthurak S Chularojanamontri L. The efficacy of UVA1 phototherapy in psoriasis: clinical and histological aspects. Photodermatol Photoimmunol Photomed. (2020) 36:21–8. doi: 10.1111/phpp.12498 31309611

[26] Arisi M Gelmetti A Focà E Rossi M Rovati C Calzavara-Pinton P. UVA1 phototherapy as a treatment option for plaque psoriasis in HIV-positive patients. Photodermatol Photoimmunol Photomed. (2020) 36:478–80. doi: 10.1111/phpp.12581, PMID: 32473061

[27] Breuer-McHam J Simpson E Dougherty I Bonkobara M Ariizumi K Lewis DE. Activation of HIV in human skin by ultraviolet B radiation and its inhibition by NFkappaB blocking agents. Photochem Photobiol. (2001) 74:805–10. doi: 10.1562/0031-8655(2001)074<0805:aohihs>2.0.co;2, PMID: 11783936

[28] Capezzera R Venturini M Bianchi D Zane C Calzavara-Pinton P. UVA1 phototherapy of Netherton syndrome. Acta Derm Venereol. (2004) 84:69–70. doi: 10.1080/00015550310015437, PMID: 15040483

[29] McGrath H Jr. Elimination of anticardiolipin antibodies and cessation of cognitive decline in a UV-A1-irradiated systemic lupus erythematosus patient. Lupus. (2005) 14:859–61. doi: 10.1191/0961203305lu2164cr, PMID: 16304728

[30] McGrath H Jr. Ultraviolet-A1 irradiation therapy for systemic lupus erythematosus [published correction appears in lupus. 2017 Dec;26(14):1573]. Lupus. (2017) 26:1239–51. doi: 10.1177/0961203317707064, PMID: 28480786 PMC5593127

[31] Mitra A Yung A Goulden V Goodfield MD. A trial of low-dose UVA1 phototherapy for two patients with recalcitrant discoid lupus erythematosus. Clin Exp Dermatol. (2006) 31:299–300. doi: 10.1111/j.1365-2230.2005.02030.x 16487126

[32] Stege H Schöpf E Ruzicka T Krutmann J. High-dose UVA1 for urticaria pigmentosa. Lancet. (1996) 347:64. doi: 10.1016/s0140-6736(96)91600-1, PMID: 8531579

[33] Gobello T Mazzanti C Sordi D Annessi G Abeni D Chinni LM. Medium- versus high-dose ultraviolet A1 therapy for urticaria pigmentosa: a pilot study. J Am Acad Dermatol. (2003) 49:679–84. doi: 10.1067/s0190-9622(03)01483-x 14512916

[34] Brazzelli V Grassi S Merante S Grasso V Ciccocioppo R Bossi G. Narrow-band UVB phototherapy and psoralen-ultraviolet a photochemotherapy in the treatment of cutaneous mastocytosis: a study in 20 patients. Photodermatol Photoimmunol Photomed. (2016) 32:238–46. doi: 10.1111/phpp.12248 27353865

[35] Schnopp C Tzaneva S Mempel M Schulmeister K Abeck D Tanew A. UVA1 phototherapy for disseminated granuloma annulare. Photodermatol Photoimmunol Photomed. (2005) 21:68–71. doi: 10.1111/j.1600-0781.2005.00145.x, PMID: 15752123

[36] Radakovic S Weber M Tanew A. Dramatic response of chronic ulcerating necrobiosis lipoidica to ultraviolet A1 phototherapy. Photodermatol Photoimmunol Photomed. (2010) 26:327–9. doi: 10.1111/j.1600-0781.2010.00543.x 21140992

[37] Beattie PE Dawe RS Ibbotson SH Ferguson J. UVA1 phototherapy for treatment of necrobiosis lipoidica. Clin Exp Dermatol. (2006) 31:235–8. doi: 10.1111/j.1365-2230.2005.02059.x, PMID: 16487100

[38] Mahnke N Medve-Koenigs K Berneburg M Ruzicka T Neumann NJ. Cutaneous sarcoidosis treated with medium-dose UVA1. J Am Acad Dermatol. (2004) 50:978–9. doi: 10.1016/j.jaad.2003.09.027 15153910

[39] Graefe T Konrad H Barta U Wollina U Elsner P. Successful ultraviolet A1 treatment of cutaneous sarcoidosis. Br J Dermatol. (2001) 145:354–5. doi: 10.1046/j.1365-2133.2001.04356.x 11531812

[40] Sakakibara N Sugano S Morita A. Ultrastructural changes induced in cutaneous collagen by ultraviolet-A1 and psoralen plus ultraviolet a therapy in systemic sclerosis. J Dermatol. (2008) 35:63–9. doi: 10.1111/j.1346-8138.2008.00417.x, PMID: 18271800

[41] Albuquerque JV Andriolo BN Vasconcellos MR Civile VT Lyddiatt A Trevisani VF. Interventions for morphea. Cochrane Database Syst Rev. (2019) 7:CD005027. doi: 10.1002/14651858.CD005027.pub5 31309547 PMC6630193

[42] Arisi M Lorenzi L Incardona P Fusano M Zanca A Rossi MT. Clinical, histological and high-frequency ultrasonographic evaluation (50 MHz) of morphea treated with ultraviolet A1 phototherapy. Clin Exp Dermatol. (2019) 44:270–6. doi: 10.1111/ced.13693 29974485

[43] Kreuter A Hyun J Stücker M Sommer A Altmeyer P Gambichler T. A randomized controlled study of low-dose UVA1, medium-dose UVA1, and narrowband UVB phototherapy in the treatment of localized scleroderma. J Am Acad Dermatol. (2006) 54:440–7. doi: 10.1016/j.jaad.2005.11.1063, PMID: 16488295

[44] Sator PG Radakovic S Schulmeister K Hönigsmann H Tanew A. Medium-dose is more effective than low-dose ultraviolet A1 phototherapy for localized scleroderma as shown by 20-MHz ultrasound assessment. J Am Acad Dermatol. (2009) 60:786–91. doi: 10.1016/j.jaad.2008.12.013 19211170

[45] Kreuter A Gambichler T Avermaete A Happe M Bacharach-Buhles M Hoffmann K. Low-dose ultraviolet A1 phototherapy for extragenital lichen sclerosus: results of a preliminary study. J Am Acad Dermatol. (2002) 46:251–5. doi: 10.1067/mjd.2002.118552 11807437

[46] Herz-Ruelas ME Barboza-Quintana O Cuéllar-Barboza A Cárdenas-de la Garza JA Gómez-Flores M. Acral bullous lichen sclerosus intolerant to UVA-1 successfully treated with narrowband ultraviolet B phototherapy. Photodermatol Photoimmunol Photomed. (2019) 35:378–80. doi: 10.1111/phpp.12478, PMID: 31062884

[47] Tognetti L Marrocco C Carraro A Conticini E Habougit C Mariotti G. UVA-1 phototherapy as adjuvant treatment for eosinophilic fasciitis: in vitro and in vivo functional characterization. Int J Dermatol. (2022) 61:718–26. doi: 10.1111/ijd.16003, PMID: 34881449 PMC9299925

[48] Grundmann-Kollmann M Behrens S Gruss C Gottlöber P Peter RU Kerscher M. Chronic sclerodermic graft-versus-host disease refractory to immunosuppressive treatment responds to UVA1 phototherapy. J Am Acad Dermatol. (2000) 42:134–6. doi: 10.1016/s0190-9622(00)90023-9, PMID: 10607334

[49] Gambichler T Schmitz L. Ultraviolet A1 phototherapy for Fibrosing conditions. Front Med. (2018) 5:237. Published 2018 Aug 27. doi: 10.3389/fmed.2018.00237, PMID: 30211165 PMC6119689

[50] Calzavara Pinton P Porta F Izzi T Venturini M Capezzera R Zane C. Prospects for ultraviolet A1 phototherapy as a treatment for chronic cutaneous graft-versus-host disease. Haematologica. (2003) 88:1169–75. PMID: 14555314

[51] Knobler R Berlin G Calzavara-Pinton P Greinix H Jaksch P Laroche L. Guidelines on the use of extracorporeal photopheresis. J Eur Acad Dermatol Venereol. (2014) 28:1–37. doi: 10.1111/jdv.12311, PMID: 24354653 PMC4291097

[52] Miziołek B Tworek M Łapczyńska E Tekielak A Kochanowska J Polak K. Utility of phototherapy in patients with systemic sclerosis: systematic review. Dermatol Ther. (2022) 35:e15478. doi: 10.1111/dth.15478, PMID: 35357072

[53] Durand F Staumont D Bonnevalle A Hachulla E Hatron PY Thomas P. Ultraviolet A1 phototherapy for treatment of acrosclerosis in systemic sclerosis: controlled study with half-side comparison analysis. Photodermatol Photoimmunol Photomed. (2007) 23:215–21. doi: 10.1111/j.1600-0781.2007.00308.x 17986056

[54] Aranegui B Jiménez-Reyes J. Morphea in Childhood: an Update. Morfea en la infancia: actualización. Actas Dermosifiliogr. (2018) 109:312–22. doi: 10.1016/j.ad.2017.06.021 29248149

[55] Kafi R Fisher GJ Quan T Shao Y Wang R Voorhees JJ. UV-A1 phototherapy improves nephrogenic fibrosing dermopathy. Arch Dermatol. (2004) 140:1322–4. doi: 10.1001/archderm.140.11.1322, PMID: 15545539

[56] Janiga JJ Ward DH Lim HW. UVA-1 as a treatment for scleredema. Photodermatol Photoimmunol Photomed. (2004) 20:210–1. doi: 10.1111/j.1600-0781.2004.00106.x, PMID: 15238100

[57] Kroft EB de Jong EM. Scleredema diabeticorum case series: successful treatment with UV-A1. Arch Dermatol. (2008) 144:947–8. doi: 10.1001/archderm.144.7.947, PMID: 18645154

[58] Thumpimukvatana N Wongpraparut C Lim HW. Scleredema diabeticorum successfully treated with ultraviolet A1 phototherapy. J Dermatol. (2010) 37:1036–9. doi: 10.1111/j.1346-8138.2010.01014.x 21083706

[59] Appelhans C Breuckmann F Bastian A Altmeyer P Kreuter A. Fibromatosis of the hand associated with EMO syndrome: a case report. BMC Dermatol. (2004) 4:17. doi: 10.1186/1471-5945-4-17, PMID: 15533248 PMC529452

[60] von Kobyletzki G Kreuter JA Nordmeier R Stücker M Altmeyer P. Treatment of idiopathic mucinosis follicularis with UVA1 cold light phototherapy. Dermatology. (2000) 201:76–7. doi: 10.1159/000018440, PMID: 10971071

[61] Gil-Villalba A de la Torre-Gomar FJ Navarro-Triviño FJ Ruiz-Villaverde R. Successful treatment of follicular mucinosis with low-dose UVA1 phototherapy. Dermatol Ther. (2022) 35:e15773. doi: 10.1111/dth.15773 35976079

[62] Amherd-Hoekstra A Kerl K French LE Hofbauer GF. Reticular erythematous mucinosis in an atypical pattern distribution responds to UVA1 phototherapy. J Eur Acad Dermatol Venereol. (2014) 28:672–3. doi: 10.1111/jdv.12247 23952909

[63] Meewes C Henrich A Krieg T Hunzelmann N. Treatment of reticular erythematous mucinosis with UV-A1 radiation. Arch Dermatol. (2004) 140:660–2. doi: 10.1001/archderm.140.6.660, PMID: 15210454

